# Ambivalence in genomic healthcare provision, cure or symptom?

**DOI:** 10.1038/s41431-023-01467-w

**Published:** 2023-10-04

**Authors:** Gabriel Watts

**Affiliations:** Sydney Health Ethics, Sydney School of Public Health, Faculty of Medicine and Health, The University of Sydney, Sydney, NSW 2006 Australia

**Keywords:** Medical ethics, Ethics

Much of genomic healthcare is simply grey. The boundaries between research and clinical work, the point where diagnostic testing becomes screening, when to report variants of uncertain significance (VUS), or secondary findings, and not least, the difficulty of determining which ethical considerations ought to take priority in such cases. Genomic healthcare professionals (GHCP) are right to be ambivalent about what is best to do, both as individuals and collectively as teams.

In their paper ‘Dealing with ambivalence in the practice of advanced genetic healthcare: towards an ethical choreography’ [1], Kuiper et al. seek to leverage such ambivalence to put forward a vision of “what ‘good’ genetic care should look like”. To do so they observe and detail the enactment of ambivalence within two large European centres for human genetics, focusing on the types of ambivalence mentioned above.

Crucially, Kuiper et al. assert that for the most part, such “ambivalence was often not made explicit or acknowledged and, therefore, the reflection on it was lacking”. Instead, where healthcare practices were observed to conflict with existing guidelines, norms, and beliefs this was “not openly contested” but rather “performatively questioned” in a “predominantly implicit” manner, which is to say, through largely unreflective acts of non- or reduced compliance. For instance, Kuiper et al. describe the typically perfunctory way that opt-in and -out options for receipt of testing information were discussed with patients, if at all, and view this negligible concern with patient informedness as an implicit assertion that “as [G]HCPs, they were best placed to know which options to choose”, in opposition to an official requirement that patient autonomy be the “starting point for the actions of caregivers”.

As Kuiper et al. see it, the passive enactment of ambivalence towards existing healthcare “structures” is problematic not because it is ambivalent (although it does “lead to confusing or mixed messages for patients” on this count) but rather because it “fail[s] to benefit from the discussion and change that acknowledged ambivalence can bring about”. In response, Kuiper et al. advocate for an “open ethical choreography” in which GHCPs are “encouraged to be open about experiences of ambivalence” and “additional voices [are] invited to contribute to understand the internal logic of the local practices and the values, interests and constrain[t]s this is built on”. This is because the inclusion of “additional voices” to the practice of genomic healthcare “will make the decision-making more informed and transparent to all and allow [a] more reflective way of approaching the future of genomic healthcare”.

I have no doubt that the three sites of ambivalence Kuiper et al. identify are widespread throughout genomic healthcare: the blurring of boundaries between research and clinical work [2], the difficulties that attend the classification of VUS and return of secondary findings [3], and the tensions between expert opinion and patient autonomy are all well-documented [4]. And I applaud their call for GHCPs to be open about experiences of ambivalence. I have my doubts, however, as to whether characterising the passive enactment of ambivalence as a failure to benefit from acknowledging ambivalence—and especially the benefit of accounting for additional voices in decision-making—is the best interpretation of such behaviour. I am ambivalent, we might say, as to whether the widespread ambivalence experienced by GHCPs is best viewed as a propaedeutic to improving professional practice, or as a symptom of healthcare structures that are insufficiently responsive to the needs of GHCPs.

Take, for instance, Kuiper et al. discussion of the difficulties facing GHCPs when considering if and how to report on variants of uncertain significance. Here, it would appear that a major cause of ambivalence, both within teams of GHCPs and within the minds of individual practitioners, is a concern that variants reported as VUS tend to accrue meanings and significance beyond what genomic healthcare specialists intend, and that the likely “reification” of VUS must be accounted for in reporting. If so, then the problem is not a lack of openness to the views of others, but rather a lack of clear reporting guidelines (as Kupier et al. note) and a need for genomics-adjacent healthcare professionals to be better trained in the interpretation of genomic results—so as to remove some of the moral burden attending reporting VUS from the shoulders of GHCPs.

The same point might be made regarding the ambivalence experienced at the boundaries of diagnostic testing and screening. Here, Kuiper et al. describe case-by-case decisions regarding the appropriateness of pursuing cascade and screening tests as serving to unreflectively widen the scope of whom next-generation sequencing (NGS) ought to be offered to. It is of course important that GHCPs be open to reflecting upon how this occurs within their own practice, and that they consider the different ethical norms that attend diagnostic testing and screening. But it would also seem that the primary problem is a lack of clear ethical guidelines regarding how to draw limits to the pursuit of diagnoses through NGS and why this ought to be done.

To be clear, I am not at all suggesting that Kuiper et al.’s analysis does not allow for such conclusions as I have drawn above. Nor do I think they are wrong to suggest that in cases like the perfunctory informed consent practices they describe, more reflection on the part of GHCPs would be a good thing. Rather, my suggestion is that, on the whole, the moral to draw from the ambivalence that they observe within the practice of genomic healthcare is not that GHCPs are failing to benefit from ambivalence to the extent that they enact such ambivalence passively, but that the moral burden of such ambivalence ought to be more evenly distributed across the healthcare system, at the risk of it ossifying into an unreflective performance of genomic care.
